# Transcriptome analysis following neurotropic virus infection reveals faulty innate immunity and delayed antigen presentation in mice susceptible to virus‐induced demyelination

**DOI:** 10.1111/bpa.13000

**Published:** 2021-07-06

**Authors:** Malgorzata Ciurkiewicz, Stefan Floess, Michael Beckstette, Maren Kummerfeld, Wolfgang Baumgärtner, Jochen Huehn, Andreas Beineke

**Affiliations:** ^1^ Department of Pathology University of Veterinary Medicine Hannover Hannover Germany; ^2^ Experimental Immunology Helmholtz Centre for Infection Research Braunschweig Germany; ^3^ Center for Systems Neuroscience University of Veterinary Medicine Hannover Hannover Germany; ^4^ Cluster of Excellence RESIST (EXC 2155) Hannover Medical School Hannover Germany

**Keywords:** antigen presentation, antiviral response, demyelination, innate immune response, mouse model, neurotropic virus, Theiler's murine encephalomyelitis virus, transcriptome analysis, viral encephalitis

## Abstract

Viral infections of the central nervous system cause acute or delayed neuropathology and clinical consequences ranging from asymptomatic courses to chronic, debilitating diseases. The outcome of viral encephalitis is partially determined by genetically programed immune response patterns of the host. Experimental infection of mice with Theiler's murine encephalomyelitis virus (TMEV) causes diverse neurologic diseases, including TMEV‐induced demyelinating disease (TMEV‐IDD), depending on the used mouse strain. The aim of the present study was to compare initial transcriptomic changes occurring in the brain of TMEV‐infected SJL (TMEV‐IDD susceptible) and C57BL/6 (TMEV‐IDD resistant) mice. Animals were infected with TMEV and sacrificed 4, 7, or 14 days post infection. RNA was isolated from brain tissue and analyzed by whole‐transcriptome sequencing. Selected differences were confirmed on a protein level by immunohistochemistry. In mock‐infected SJL and C57BL/6 mice, >200 differentially expressed genes (DEGs) were detected. Following TMEV‐infection, the number of DEGs increased to >700. Infected C57BL/6 mice showed a higher expression of transcripts related to antigen presentation via major histocompatibility complex (MHC) I, innate antiviral immune responses and cytotoxicity, compared with infected SJL animals. Expression of many of those genes was weaker or delayed in SJL mice, associated with a failure of viral clearance in this mouse strain. SJL mice showed prolonged elevation of MHC II and chemotactic genes compared with C57BL/6 mice, which presumably facilitates the induction of chronic demyelinating disease. In addition, elevated expression of several genes associated with immunomodulatory or –suppressive functions was observed in SJL mice. The exploratory study confirms previous observations in the model and provides an extensive list of new immunologic parameters potentially contributing to different outcomes of viral encephalitis in two mouse strains.

## Graphical abstract

Comparative transcriptome analysis of two mouse strains with different susceptibility to chronic viral CNS infection shows marked differences in the expression of immune genes. Mice susceptible to virally induced demyelinating disease have a delayed or weaker upregulation of MHC I, NK cell responses, and innate immune genes but show increased expression of MHC II and chemotactic genes.

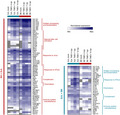

## 1 INTRODUCTION

Many viral infections of the central nervous system (CNS) cause acute or delayed neuropathology with potential severe consequences for the structural and functional integrity of an organ system with limited regenerative capacity (1, 2, 3). Besides directly damaging infected cells, viruses can elicit pathologic neuroinflammation and trigger the immune system to recognize self‐antigens and cause autoimmune responses. In fact, some autoimmune diseases, such as multiple sclerosis (MS) or Guillan‐Barré Syndrome, are suspected to be initiated by virus infection (4, 5). The outcome of viral encephalitis varies among individuals and is influenced by many factors, including the genetic background of the host (6, 7). Experimental infection of mice with Theiler's murine encephalomyelitis virus (TMEV, Theilervirus) is an interesting model to study different sequelae of neurotropic virus infection (8, 9). TMEV is a single‐stranded RNA virus belonging to the *Picornaviridae*, a family including many neuropathogenic viruses of humans and other mammals. Experimental intracerebral infection of laboratory mice with low‐virulent TMEV strains, such as BeAn and DA, uniformly causes a transient polioencephalitis with predominant infection of neurons in the hippocampus, cortex, and thalamus (10). In immunocompetent animals, this phase is usually not associated with obvious neurologic impairment or lethality. The long‐term consequences of acute infections are highly dependent on the genetic background of the host (11, 12). In SJL mice, infection leads to a lifelong persistence, predominantly in glial cells and macrophages within the white matter of the spinal cord (13, 14, 15, 16). Persistent infection is associated with TMEV‐induced demyelinating disease (TMEV‐IDD), which is characterized by chronic demyelination, inflammation, and axonal damage in the spinal cord and leads to a progressive decline of motor function. The disease features a Th1‐mediated, delayed‐type hypersensitivity response to viral epitopes and possibly also antibody‐ and cell‐mediated autoimmunity directed against myelin and oligodendrocyte antigens (8, 9, 17, 18). Because of several similarities regarding pathogenesis, clinical course and histologic lesions, TMEV‐IDD represents a valuable model for the progressive form of MS (17, 18, 19, 20). In contrast to the SJL strain, C57BL/6 (B6) mice clear the virus within the first weeks of infection and do not develop TMEV‐IDD. However, depending on the virus strain and the dose of infection, B6 mice show marked hippocampal neuronal damage and spontaneous behavioral seizures (21, 22, 23, 24). A proportion of acutely seizing mice further develops a permanently altered seizure threshold and epilepsy (24, 25). Hippocampal damage is also associated with behavioral abnormalities, such as cognitive impairment and anxiety‐like behavior (26, 27). Therefore, TMEV‐infection of B6 mice has recently gained considerable importance as a viral model for epilepsy and other neurologic long‐term effects of transient viral encephalitis (8, 19).

The exact mechanisms underlying differences in disease course between SJL and B6 mice are not fully understood. Susceptibility to virus persistence and TMEV‐IDD is a quantitative, multigenic trait (28, 29). Among the susceptibility genes, the major histocompatibility complex (MHC) I gene *H2‐D* plays a major role in protection from virus‐persistence and chronic disease. Mice with the H‐2D^b^ haplotype (B6, B10) are fully resistant, and animals with the H‐2D^s^ haplotype (SJL) are fully susceptible (30, 31, 32). B6 mice generate a protective, H2‐D‐restricted CD8^+^ T cell response directed against the dominant virus capsid peptide VP3_159‐166_ (29, 30, 32, 33, 34). By contrast, only H2‐K‐restricted virus‐specific CD8^+^ T cells are present in SJL mice, and those are directed against viral epitopes distinct from B6 mice (35). Moreover, the absolute number of CD8^+^ T cells detected in the CNS during very early stages of the infection is higher in B6 than in SJL mice (36). One possible explanation for a delayed homing of cytotoxic T lymphocytes (CTL) into the CNS in SJL mice is a low expression of H2‐K molecules in the CNS in this strain (35, 37). In addition to differences in the CTL compartment, a skewed polarization of the CD4^+^ T cell response has been linked to susceptibility to TMEV‐IDD in SJL mice. For instance, SJL mice show an early expansion of Foxp3^+^ regulatory T cells (Tregs), which potentially dampen antiviral CD8^+^ T cell responses (38, 39, 40, 41). Moreover, an unfavorable Th1/Th17 ratio with elevated Th17 numbers in SJL mice contributes to virus persistence, for example by inhibiting apoptosis of infected cells (42, 43, 44, 45, 46, 47). Antigen‐presenting cells (APCs) derived from the bone marrow of SJL mice support vigorous TMEV replication and promote pathogenic Th17 differentiation, probably by inducing higher levels of IL‐6 and other cytokines. By contrast, APCs derived from B6 or B6. S mice are less permissive to TMEV replication and induce stronger protective Th1 responses (44, 45). Hence, genetically programed innate immune response patterns activated within infected cells also determine the polarization of adaptive immune responses and thus influence the final outcome of TMEV‐infection (8, 48). The genes responsible for differential permissiveness of APCs to support TMEV replication have not been elucidated, yet.

The aim of the present study was to determine transcriptomic differences occurring in the brain during the course of an acute neurotropic virus infection in SJL and B6 mice, to put them in the context of the existing data, and to identify unknown genes and pathways potentially associated with the outcome of TMEV‐infection. Pairwise comparisons of expression between SJL and B6 mice at different timepoints after infection revealed marked absolute differences in the expression of several immune response genes, including classical and nonclassical MHC genes, innate antiviral genes as well as complement and chemokine transcripts. An additional time course analysis indicated that some of these genes also show significantly different expression kinetics over the course of acute brain infection. In brief, SJL mice showed a delayed and weaker upregulation of MHC I molecules and several innate antiviral genes, which could be factors contributing to inefficient virus clearance. In addition, a stronger and prolonged expression of MHC II and several chemotactic genes was observed in SJL animals during late polioencephalitis, indicating prolonged neuroinflammation. The latter observation could reflect an early event in the induction of hypersensitivity and autoimmune responses. The results provide a basis for further experiments targeting protective molecular mechanisms in viral encephalitis.

## 2 MATERIALS AND METHODS

### 2.1 Animals and virus infection

Female SJL/JHanHsd (SJL) and C57BL/6JOlaHsd (B6) mice were purchased from Harlan Winkelmann, Borchen, Germany. At the age of 5 weeks, mice of each strain were anesthetized with medetomidine (0.5 mg/kg; Domitor, Pfizer) and ketamine (100 mg/kg, ketamine 10%; WDT eG), and inoculated into the right cerebral hemisphere with 1.63 x 10^6^ plaque forming units of TMEV BeAn diluted in 20 µl Dulbecco's modified Eagle medium (PAA Laboratories) with 2% fetal calf serum and 50 µg/kg gentamycin. Control animals were inoculated with 20 µl of vehicle only (mock). Animals were assigned randomly into TMEV or the mock group. Groups of six animals were killed at 4, 7, 14, 28, 56, or 98 days post infection (dpi). Brain and spinal cord tissue were removed immediately after death, and selected parts were either fixed in 10% formalin for histology, immunohistochemistry, and *in situ* hybridization (caudal half of cerebrum and cervical, thoracic, and lumbar spinal cord segments) or embedded in OCT® embedding compound (Sakura Finetek Europe B. V), snap frozen, and stored at −80℃ until use for RNA extraction (cranial half of cerebrum).

### 2.2 Histology

After formalin fixation for 24 h, brain and spinal cord samples were embedded in paraffin and 2‐µm thick, transversal, serial sections were prepared. Inflammation was assessed on hematoxylin and eosin‐stained sections using a semiquantitative scoring system as described previously (10, 38). Briefly, the two‐tiered system included grading of perivascular infiltrates (0 = no changes, 1 = one layer; 2 = 2 to 3 layers; 3 = more than 3 layers of perivascular inflammatory cells) and intraparenchymal hypercellularity/gliosis (0 = no change, 1 = 1–25 cells, 2 = 26–50 cells, 3 = >50 cells). In the cerebrum, separate scores were given to distinct anatomical regions (meninges, cortex, corpus callosum, hippocampus, thalamus/hypothalamus, and ependyma/periventricular region of the third and lateral ventricles). The total score reflects the sum of scores in both categories in all regions. In the spinal cord, inflammation was assessed separately in the meninges, white and grey matter of one section each from the cervical, thoracic and lumbar region, and a sum of all scores was calculated (10). Demyelination of the spinal cord was assessed semiquantitatively on *Luxol‐fast‐blue‐cresyl‐violet* (LFB‐CV)‐stained sections by estimating the percentage of the affected white matter in one cervical, thoracic, and lumbar section, respectively (0 = no change, 1 = 25%, 2 = 25%–50% and 3 = 50%–100%) (49).

### 2.3 Immunohistochemistry

TMEV‐, natural cytotoxicity triggering receptor 1 (NCR1/ NKp46)‐, carcinoembryonic antigen‐related cell adhesion molecule 1 (CEACAM1)‐, chemokine (C‐X‐C motif) ligand 13 (CXCL13)‐ and cathepsin E (CTSE)‐specific immunohistochemistry was performed on formalin‐fixed and paraffin‐embedded tissue as described before (38, 50). With the exception of TMEV, antigen retrieval was performed by boiling the slides for 20 min in citrate buffer. Slides were incubated with the following primary antibodies: polyclonal rabbit anti‐TMEV (1:2000) (50), polyclonal goat anti‐mouse NKp46/NCR1 (R&D Systems, AF2225, 1.5 µg/ml), polyclonal goat anti‐mouse CXCL13/BLC/BCA‐1 IgG (R&D Systems, AF470, 1.5 µg/ml), polyclonal goat anti‐mouse cathepsin E IgG (R&D Systems, AF1130, 1.5 µg/ml), and polyclonal sheep anti‐mouse CEACAM‐1/CD66a IgG (AF6480, R&D Systems, 2.5 µg/ml). Primary antibodies were incubated for 1.5 h at room temperature (TMEV) or at 4℃ overnight (all other). For negative controls, specific isotype controls or sera were used. Biotinylated secondary antibodies (Vector Laboratories, 1:200) were incubated for 45 min at room temperature. Absolute numbers of TMEV‐, NCR1‐, and CXCL13‐immunolabeled cells were counted in entire cross sections of the forebrain. CEACAM1 and CTSE antigen was quantified on digitalized slides by densitometry using the analySIS® 3.2 software, covering the entire transversal section of the forebrain. TMEV‐positive cells were additionally enumerated in one section each from the cervical, thoracic, and lumbar spinal cord.

### 2.4 *In situ* hybridization

*In situ* hybridization to detect TMEV‐specific RNA was performed as described before (10, 51). In summary, a PCR product was generated from a TMEV‐infected baby hamster kidney cell line by RT‐PCR using the following primers: forward: 5′‐GACTAATCAGAGGAACGTCAGC‐′3 and reverse: 5′‐GTGAAGAGCGGCAAGTGAGA‐′3, homologous to the base pair 193 to 322 of the sequence for BeAn 8386 (52). The obtained PCR product was cloned into PCR 4‐TOPO plasmid vector and amplified in DH5 α‐T1® cells (TOPO TA Cloning Kit for sequencing; Invitrogen). The plasmid was sequenced (SEQLAB, sequence is accessible under GenBank accession number: AY618571). *In vitro* transcription was carried out according to manufacturer's instructions with DIG‐RNA‐labeling mix and T3‐ and T7‐RNA‐polymerases (Roche Diagnostics, Mannheim, Germany). Brain and spinal cord tissue sections were dewaxed in xylene, hydrated and washed, followed by proteolyses (5 µg/ml proteinase K; Roche Diagnostics), acetylation, and prehybridization. Hybridization was performed overnight in a moist chamber at 52° with a probe concentration of 200 ng/ml. Detection was performed with an anti‐DIG‐antibody conjugated with alkaline phosphatase (1:200; Roche Diagnostics, Mannheim, Germany) and the substrates nitroblue tetrazolium chloride (NBT; Sigma‐Aldrich), and 5‐bromo‐4‐chloro‐3‐indolyl phosphate (BCIP, X‐Phosphate; Sigma‐Aldrich). The absolute numbers of labeled cells were counted in different regions of the forebrain and spinal cord (see Section 2.2).

### 2.5 Statistical analysis (histology, immunohistochemistry, and *in situ* hybridization)

Histology, immunohistochemistry, and *in situ* hybridization results were analyzed by multiple Mann–Whitney U‐tests using SPSS for windows (SPSS Inc.). For the analysis of correlation between TMEV‐antigen and –RNA positive cells, a Spearman rank correlation coefficient (R_s_) was applied using SPSS. In general, a *p*‐value of <0.05 was considered statistically significant. Graphs were created with GraphPad PrismVR (GraphPad Software).

### 2.6 RNA‐based next generation sequencing (RNA‐seq)

For a comparative analysis of the early transcriptional changes in TMEV‐IDD susceptible (SJL) and resistant (B6) mice, 4–5 TMEV‐infected animals of each strain were selected from three early time points post infection (4, 7, and 14 dpi). Three mock‐infected B6 and three mock‐infected SJL animals, sacrificed at 7 dpi, served as controls. RNA was isolated from frozen cerebral tissue using an Omni´s PCR Tissue Homogenizing Kit (Süd‐Laborbedarf GmbH) and QIAzolTM Lysis Reagent and RNeasy® Mini Kit (Qiagen GmbH) according to the manufacturer's instructions. The quality and integrity of the isolated RNA were assessed using Agilent Technologies 2100 Bioanalyzer (Agilent Technologies). Purification of poly‐A containing mRNA was performed using poly‐T oligo attached magnetic beads (Illumina). Subsequently, mRNA was used for library preparation using the Script Seq v2 Library preparation kit (Illumina). Sequencing was carried out on Illumina HiSeq2500 using 50 bp single‐read. The sequenced libraries were assessed for read quality with *FastQC* (http://www.bioinformatics.babraham.ac.uk/projects/fastqc). Quality assessment showed neither insufficient read quality, nor nucleotide frequency biases introduced by primer contamination. Therefore, libraries were directly aligned to mouse reference genome (assembly: GRCm38) using splice junction mapper *Tophat2* v1.2.0 with default parameterization (53). Reads aligned to annotated genes were quantified with htseq‐count (http://www‐huber.embl.de/users/anders/HTSeq) program and RPKM (reads per kilobase million) normalized values were computed from raw gene counts. Principal component analysis (PCA) of the log2 transformed, scaled, and mean centered RPKM values was performed using base functions *scale* and *prcomp* from the statistical data analysis framework R. First, a pairwise comparison of gene expression in SJL and B6 mice was performed for each condition (mock and TMEV), and timepoint following TMEV infection (4, 7, and 14 dpi) to detect which genes showed the highest absolute differences between strains. Read counts served as input to DESeq2 (54), and the list of differentially expressed genes (DEGs) was filtered with an absolute log2fold change (FC) cut‐off of at least 1.5 and a *p*‐value cut‐off, corrected for multiple testing, of at most 0.05. A heatmap depicting the log2FC of DEGs (Figure 3) was generated, and a k‐means clustering with k = 10 was performed to group genes with a similar expression pattern. In addition to pairwise comparisons between SJL and B6 mice, a time course analysis was performed employing the likelihood ratio test from the DESeq2 package in R (cut‐off of *p* < 0.01) to detect strain‐specific effects of TMEV‐infection on gene expression over time.

### 2.7 Functional annotation

To obtain further information about the biology of DEGs obtained by pairwise comparisons and time course analysis, functional annotation and overrepresentation analysis was performed using the application *WEB‐based GEne SeT AnaLysis Toolkit* (*WebGestalt 2019*) (55, 56). Overrepresented gene ontology (GO) terms in the category biological process were determined for DEG sets with a minimum of three genes for a category, and an FDR cut‐off of <0.05, using Benjamini‐Hochberg method for multiple test adjustment.

## 3 RESULTS

### 3.1 SJL and B6 mice infected with Theiler's murine encephalomyelitis virus show transient viral encephalitis with comparable viral load and inflammation in the brain

**FIGURE 1:**
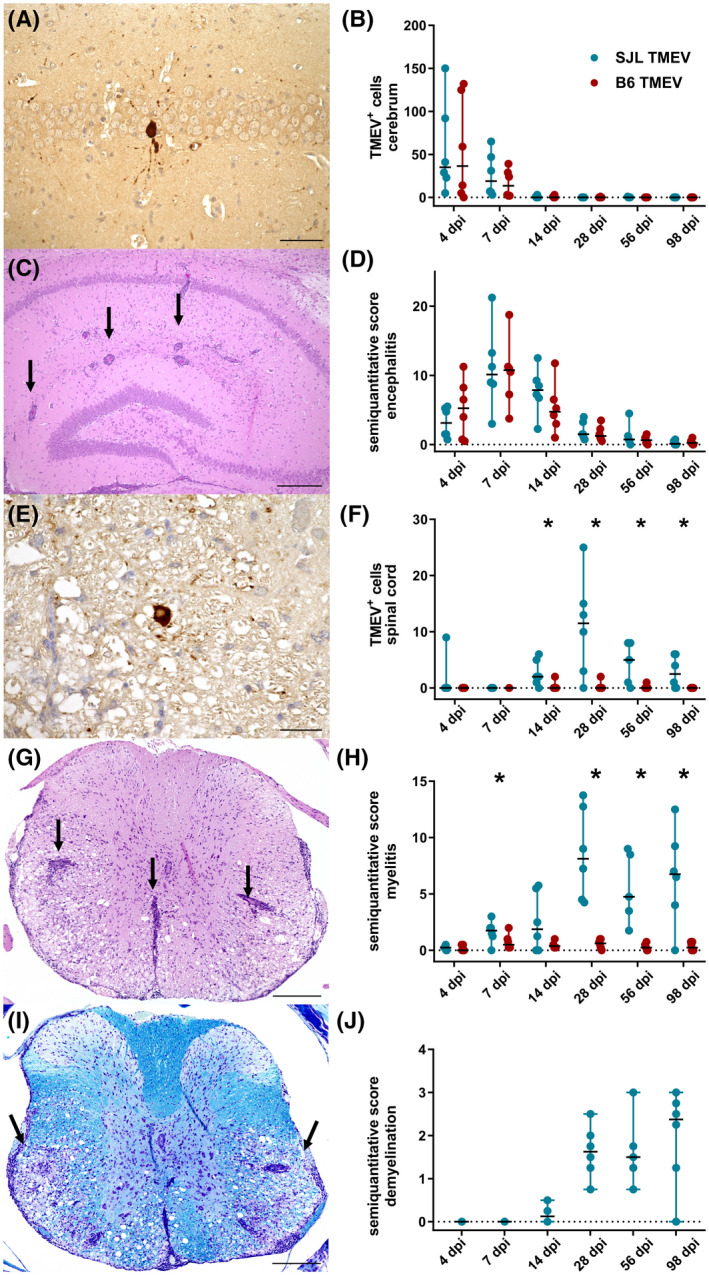
Viral load, inflammation, and demyelination in Theiler's murine encephalomyelitis (TMEV)‐infected SJL and B6 mice. (A) TMEV antigen (brown signal) in a hippocampal pyramidal neuron of an SJL mouse at 4 days post infection (dpi). (B) Quantification of viral antigen in the cerebrum by immunohistochemistry (IHC). (C) Perivascular cuffs (arrows) and hypercellularity in the hippocampus of a TMEV‐infected SJL mouse at 7 dpi. Hematoxylin eosin staining. (D) Semiquantitative scores of hippocampal inflammation. (E) TMEV antigen (brown signal) in the spinal cord white matter of an SJL mouse at 56 dpi. (F) Quantification of viral antigen in the spinal cord by IHC. (G) Perivascular infiltrates in the spinal cord white matter and median fissure (arrows) of an SJL mouse at 56 dpi. (H) Semiquantitative myelitis scores. (I) Bilateral loss of myelin (reduced blue staining) in ventral funiculi of a TMEV‐infected SJL mouse at 56 dpi (luxol fast blue‐cresyl violet). (J) Demyelination score in TMEV‐infected SJL mice. B, D, F, H, J: lines show median and range. *Significant difference (Mann–Whitney U‐Test, *p* < 0.05) n = 5–6 animals/group and time point. Scale bars: 25 µm (A, E) and 200 µm (C, G, I)

Five‐week old SJL and B6 mice were infected intracerebrally with TMEV or vehicle (mock) and groups of animals were sacrificed at 4, 7, 14, 28, 56, and 98 dpi. Viral load and pathological lesions within the cerebrum and spinal cord were evaluated using histology, immunohistochemistry, and *in situ* hybridization. In the cerebrum, TMEV antigen was detected in cells with a neuronal morphology in the hippocampus, cortex, thalamus, and hypothalamus (Figure 1A). Both mouse strains showed a transient infection with highest numbers of TMEV^+^ cells at 4 and 7 dpi (Figure 1B).

Subsequently, the cerebral viral load markedly decreased and only a few immuno‐labeled cells were found in individual animals at 14, 28, and 56 dpi, while no TMEV antigen was detectable at 98 dpi. Positive‐stranded viral RNA, visualized by *in situ* hybridization, generally co‐localized with TMEV antigen. Absolute numbers of cells containing (‐)RNA were lower in most cases, indicating a partly restricted viral replication (*data not shown*). The results obtained by immunohistochemistry and *in situ* hybridization showed a significant correlation (Table S1). Interestingly, no differences in viral load were observed in the cerebrum between SJL and B6 mice at any time point with any method (*p* > 0.228 for antigen, *p* > 0.261 for (+)RNA and *p* > 0.624 for (−)RNA). No virus was detected in cerebral tissue of mock‐infected animals. Cerebral inflammation was characterized by mononuclear, perivascular infiltrates and hypercellularity/gliosis (Figure 1C). In both mouse strains, encephalitis peaked at 7 dpi and decreased continuously thereafter (Figure 1D). Similar to the viral load, statistical analysis yielded no significant differences in the degree of cerebral inflammation between SJL and B6 mice. Apart from an occasional, mild gliosis at the injection site, no inflammatory changes were observed in mock‐infected animals.

As expected, major differences were observed in the spinal cord during late TMEV‐infection. Immunohistochemistry and *in situ* hybridization revealed a chronic infection in SJL mice, localized mainly in the white matter of the spinal cord (Figure 1E,F). In B6 mice, 1–2 TMEV‐antigen positive cells were found in one animal each at 14, 28, and 56 dpi, but no viral RNA was detected with *in situ* hybridization, resulting in significant differences in viral load in comparison to SJL mice (*p*‐values: 0.317, 1.000, 0.031, 0.013, 0.028, and 0.022 for immunohistochemistry, and 0.003, 0.028, 0.022 for *in situ* hybridization at 4, 7, 14, 28, 56, and 98 dpi, respectively). In association with the viral load, leukomyelitis was present throughout the investigation period in infected SJL mice (Figure 1G,H), while B6 mice only rarely showed a mild inflammation (*p*‐values: 0.138, 0.020, 0.146, 0.004, 0.006, and 0.035 at 4, 7, 14, 28, 56, and 98 dpi, respectively). Progressive demyelination, characterized by vacuolization and a loss of LFB‐staining intensity of the white matter, was exclusively observed in TMEV‐infected SJL mice (Figure 1I,J).

In summary, results were consistent with the typical course of TMEV‐infection of SJL and B6 mice with transient polioencephalitis observed in both mouse strains and persistent spinal cord infection of SJL mice.

### 3.2 Direct comparison of cerebral transcriptomes of SJL and B6 mice shows that most differences occur at early stages of virus infection

The susceptibility of SJL mice to TMEV‐IDD is associated with the inability to clear the virus from the CNS and with a spread of TMEV from the brain into the spinal cord. In resistant mice, virus is usually eliminated completely during the acute encephalitis phase due to a rapid and strong antiviral immune response. In order to gain more insight into the differences in gene expression in this early infection stage, transcriptome analysis of cerebral tissue obtained from mock‐ and TMEV‐infected SJL and B6 mice was performed.

**FIGURE 2:**
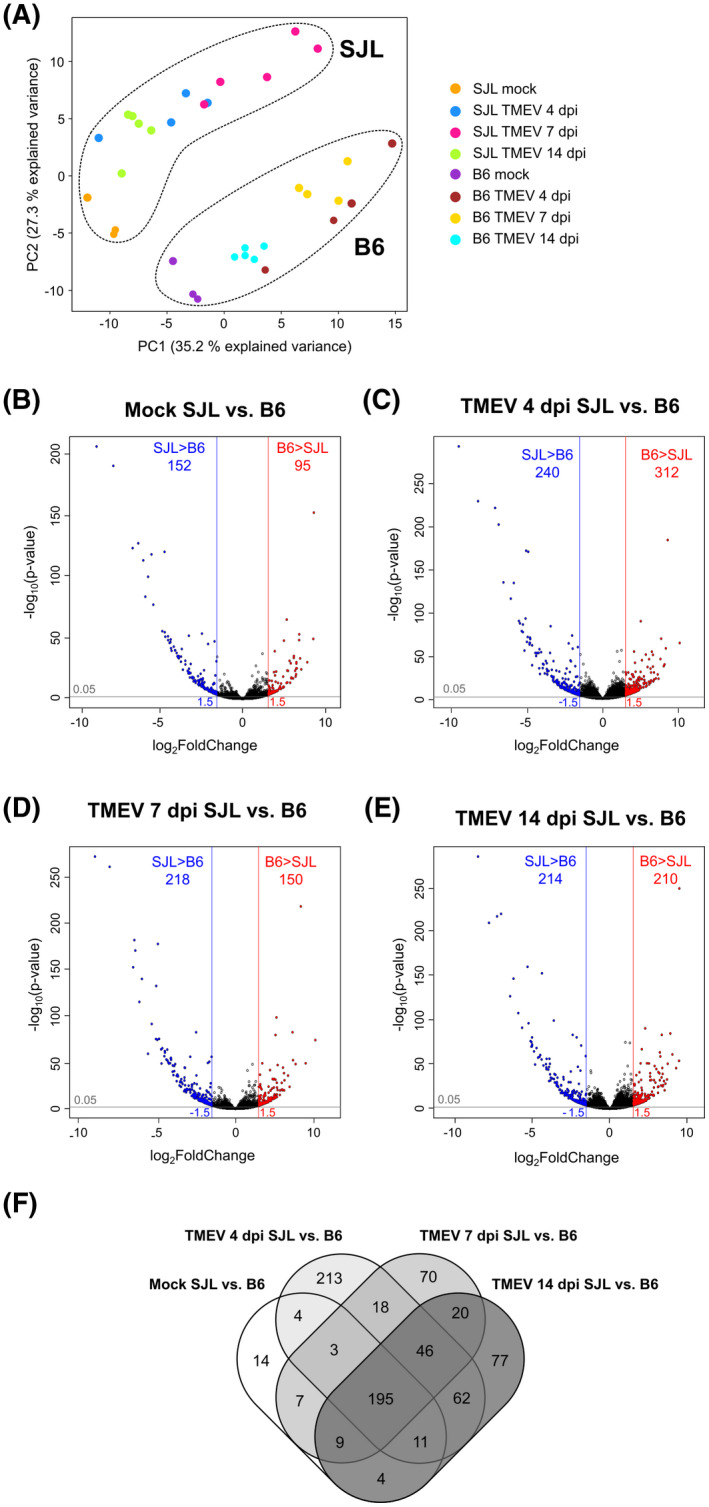
Comparison of cerebral gene expression between B6 and SJL mice following mock‐ or Theiler's murine encephalomyelitis virus (TMEV) infection. (A) Quality control of RNA‐seq data with principal component analysis (PCA). Biplot of PCs of scaled and centered, rlog transformed, normalized counts. (B–E) Volcano plots obtained by pairwise comparisons of DESeq2 normalized counts of mock‐ or TMEV‐infected SJL and B6 mice (reference: SJL). Cut‐off for differential expression was set at │log2fold change (FC)│ > 1.5 and a corrected *p*‐value of <0.05. Genes with a higher expression in SJL mice (log2FC < −1.5) are shown in blue and with a higher expression in B6 mice (log2FC > 1.5) in red. (F) Venn diagram depicting number of overlapping differentially expressed genes in mock‐ or TMEV‐infected animals at different time points

Principal component analysis (PCA) showed a clear separation of brain samples derived from SJL and B6 mice (Figure 2A). Samples obtained at 14 dpi localized closer to those of mock‐infected animals, in line with the observed declining viral load and inflammation at that time point. DESeq2 normalized counts were compared between SJL and B6 mice (reference: SJL) following mock‐ or TMEV‐infection and the cut‐off for differential expression was set at │log2fold change (FC)│ > 1.5 and a corrected *p*‐value (padj) of <0.05. The numbers of DEGs detected at the different time points are depicted in Figure 2B‐F. Comparison of mock‐infected SJL and B6 mouse transcripts revealed 247 DEGs. Ninety‐five of those showed a higher (log2FC > 1.5) and 152 a lower expression (log2FC < −1.5) in B6 compared with SJL mice, respectively (Figure 2B, Table S2). Following TMEV‐infection, the number of DEGs increased to 552 at 4 dpi, 368 at 7 dpi, and 424 at 14 dpi (Figure 2C–E, Table S2). In total, 753 genes were differentially expressed between SJL and B6 mice at one or more time points. Most differences following infection were caused by unequal upregulations of transcription compared to mock‐infected animals, while only a few genes were downregulated upon infection (Figure S1). The majority of DEGs detected under noninfectious conditions was also differentially expressed between SJL and B6 mice following TMEV‐infection, except for 14 genes (Figure 2F). The highest number of DEGs detected at one exclusive time point was observed at 4 dpi. At that time point, more genes showed a higher expression in TMEV‐infected B6 compared with TMEV‐infected SJL mice, while the opposite was true for 7 and 14 dpi. Thus, most transcriptomic differences following TMEV‐infection already occur in the early phase, prior to the peak of inflammation.

### 3.3 SJL and B6 mice show different expression levels of genes involved in immune responses and antigen presentation under noninfectious and infectious conditions

Among the 242 DEGs detected by direct comparison of brain transcripts from mock‐infected SJL and B6 mice, several genes were related to immune responses. Functional annotation and overrepresentation analysis using GO terms in the category *biological process* revealed a significant overrepresentation of the terms *immune response, antigen processing and presentation of peptide antigen and T cell‐mediated cytotoxicity* (Table 1, for details see Table S3). The DEGs included six genes of the MHC complex: one MHC class Ia (*H2‐D1*) and three class Ib genes (*H2‐Q2, H2‐T22, H2‐T24*) showed a higher expression in B6 compared with SJL mice, while transcripts of one class Ib gene (*H2‐T10*) and one class II gene (*H2‐Ob*) were increased in SJL mice (Table S2). Besides MHC genes, the DEG list contained several genes related to immune responses, which have not previously been linked to resistance to TMEV‐infection. For example, mock‐infected B6 mice showed a higher expression of *ribonuclease L (Rnasel), carcinoembryonic antigen‐related cell adhesion molecule 1 (Ceacam1), apolipoprotein A‐II (Apoa2), and tripartite motif‐containing 12A (Trim12a)*, compared with mock‐infected SJL animals. By contrast, mock‐infected SJL mice showed a higher expression of *chemokine (C‐C motif) ligand 17 (Ccl17), atypical chemokine receptor 4 (Ackr4), mannan‐binding lectin serine peptidase 2 (Masp2), and interleukin‐9 receptor (Il9r)* compared with mock‐infected B6 mice. No transcripts of synuclein alpha (*Snca*) were detected in B6 mice, which is expected due to the known deletion of the *Snca* locus in the used C57BL/6J‐OlaHsd mouse strain (57).

**TABLE 1:** Overrepresentation analysis of gene sets with differential expression between mock‐ or TMEV‐infected SJL and B6 mice

Gene set	GO term	FDR
DEGs only mock‐infected animals (n = 247)	Immune response	0.0497
	T cell‐mediated cytotoxicity	0.0358
	Antigen processing and presentation of peptide antigen	0.0497
DEGs only TMEV‐infected animals (n = 506)	Immune response	<0.0001
	Innate immune response	0.0001
	Immune effector process	0.0019
	Antigen processing and presentation	0.0256
	Leukocyte‐mediated cytotoxicity	0.0262
	Chemokine‐mediated signaling pathway	0.0442
	Response to cytokine	0.0078
	Response to interferon beta	0.0019
	Cell adhesion	0.0189
DEGs in cluster 1 (n = 277)	Immune response	0.0045
	Innate immune response	0.0045
	Immune effector process	0.0161
	Natural killer cell activation	0.0085
	Response to interferon‐beta	0.0066
	Positive regulation of leukocyte‐mediated cytotoxicity	0.0173
DEGs in cluster 2 (n = 93)	Immune response	0.0335
	Negative regulation of secretion	0.0398
	T cell mediated cytotoxicity	0.0158
	Antigen processing and presentation of endogenous peptide antigen	0.0232
	Antigen processing and presentation of endogenous peptide antigen via MHC class Ib	0.0200
DEGs in cluster 4 (n = 157)	Chemokine‐mediated signaling pathway	0.0063

Note

**FIGURE 3:**
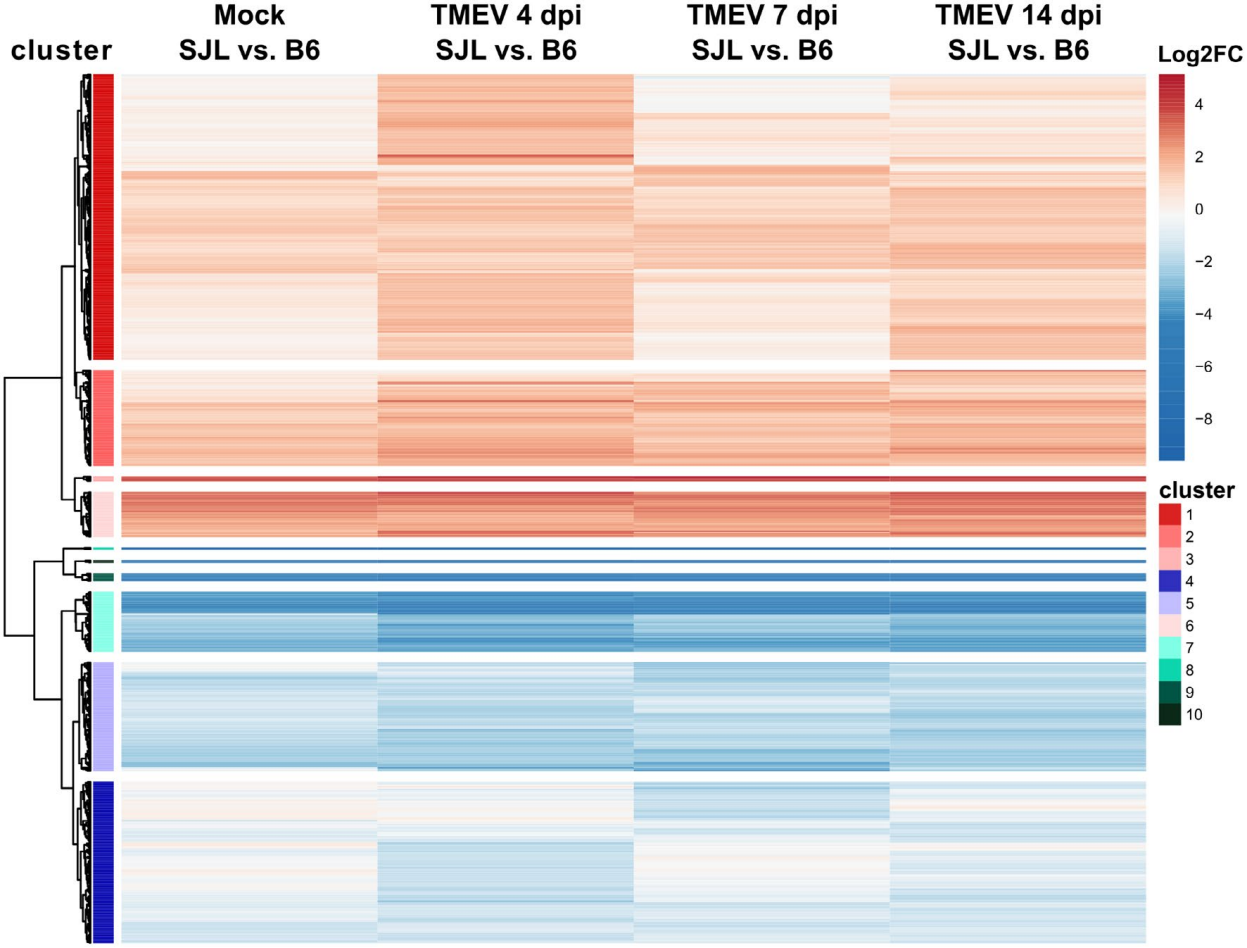
Heatmap depicting the differences in expression of 753 differentially expressed genes (DEGs) detected by pairwise comparisons of mock or TMEV‐infected SJL and B6 mice. The color scheme depicts log2 fold changes (log2FC) obtained by pairwise comparisons of DESeq2 normalized counts of mock‐ or TMEV‐infected SJL and B6 mice (reference: SJL). Cut‐off for differential expression was set at │log2fold change (FC)│ > 1.5 and a corrected *p*‐value of <0.05. Genes with a higher expression in B6 mice (log2FC > 0) are shown in red and with a higher expression in SJL mice (log2FC < 0) in blue. The DEGs were grouped by k‐means clustering (k = 10) to visualize similar expression patterns. Clusters 1–3 and 6 contain genes with higher expression in B6 mice and clusters 4, 5 and 7–10 genes with a higher expression in SJL mice

GO term: significantly enriched gene ontology terms in the category biological process were determined with WebGestalt (see Section 2). The table contains a selection of nonredundant GO‐terms related to immune responses. The full list of overrepresented terms is given in Table S3. No. of genes: total number of genes annotated to the respective GO term. FDR: false discovery rate. Values below or equal to 0.05 were considered significant. Cluster numbers refer to the gene clusters with a similar expression pattern as displayed in Figure 3.

Following TMEV infection, additional 506 genes showed differential expressions between SJL and B6 mice. Functional annotation revealed that many GO‐terms related to immune responses were overrepresented in this gene set as well (Table 1). Functional categories included the terms *immune response, innate immune response, antigen processing and presentation, leukocyte‐mediated cytotoxicity, chemokine‐mediated signaling pathway, response to cytokine, and response to interferon beta*.

### 3.4 Infected B6 mice show earlier and stronger upregulation of genes related to antigen presentation, natural killer cell activation, and innate antiviral immune responses

To obtain more information about the expression profiles of DEGs determined by pairwise comparison of SJL and B6 mice over the course of TMEV infection, a heatmap depicting the log2FC was generated, and genes with similar patterns were grouped by unsupervised k‐means clustering (Figure 3). The mean log2FCs for each cluster are depicted in Figure S2. A k = 10 was chosen to obtain clusters with sufficient gene numbers to perform an enrichment analysis. Clusters 1 to 3 and cluster 6 contained genes which in most conditions showed higher absolute expression values in B6 mice compared with SJL mice. In cluster 1 and 2, the mean log2FC was below the cut‐off for differential expression in mock‐infected animals, but markedly increased following TMEV infection, with a peak at 4 dpi. Overrepresentation analysis revealed that many genes in cluster 1 were related to *innate immune responses, natural killer cell activation, cytotoxicity and response to interferon beta* (Table 1). In cluster 2, genes belonging to the categories *antigen processing and presentation via MHC class Ib and T cell‐mediated cytotoxicity* were overrepresented. Genes in clusters 3 and 6 were already differentially expressed in mock‐infected animals (mean log2FC > 1.5), and the differences increased only mildly following TMEV infection. Due to the low number of genes in these clusters (n = 5 and 44, respectively), no significantly enriched GO terms were detected.

**FIGURE 4:**
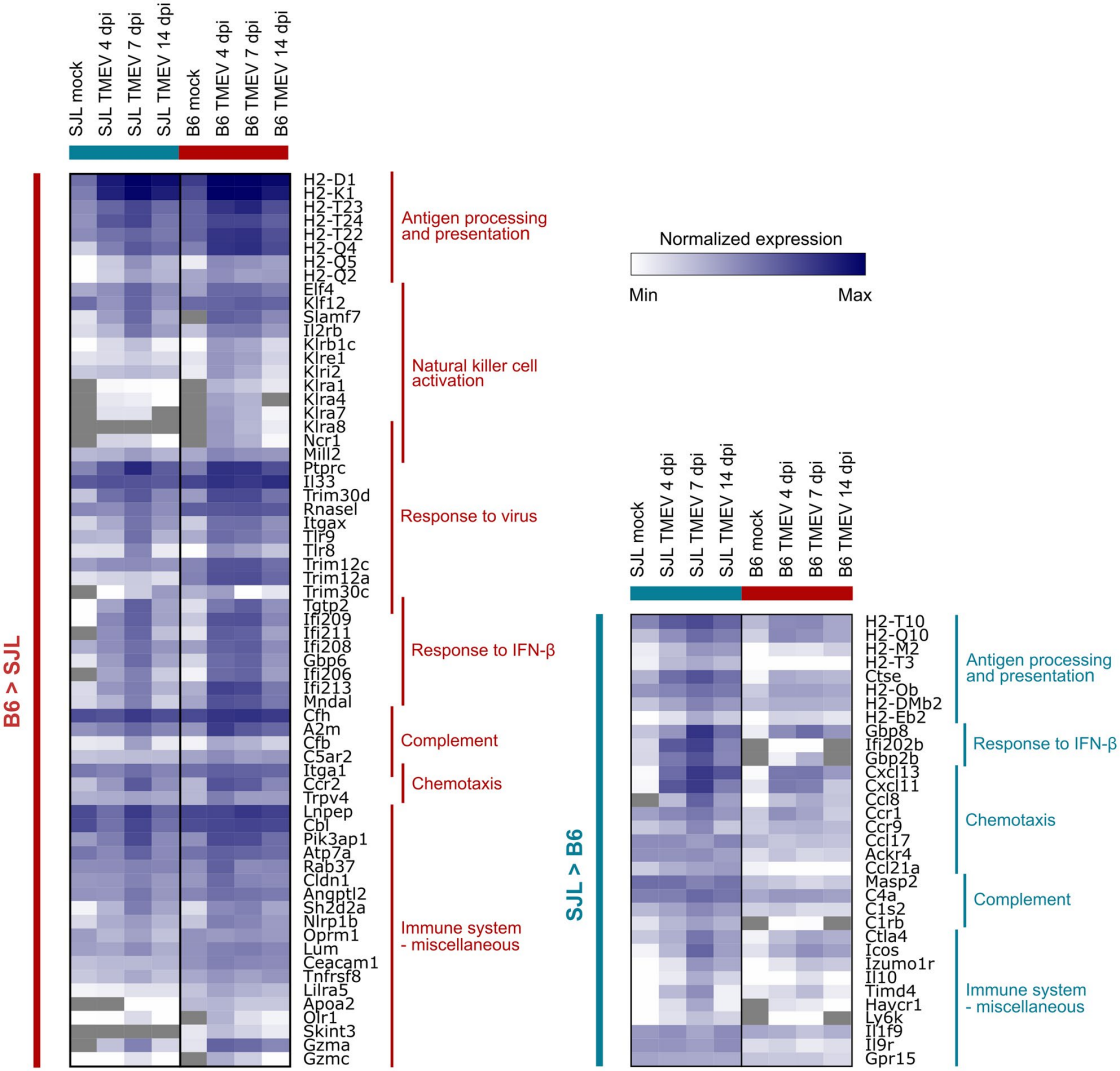
Heatmap showing expression of selected differentially expressed genes involved in immune responses. The color scheme indicates the group mean of log 2‐transformed, DESeq2 normalized counts. The left panel shows genes with a higher expression in B6 mice and the right panel genes with higher expression in SJL mice. Selected gene ontology terms associated with groups of genes are indicated on the right side of each panel

The fold changes derived by pairwise comparisons of SJL and B6 mice only show the absolute difference in expression at a given timepoint, but do not indicate whether the changes are caused by up‐ or downregulation of genes in the respective mouse strain. Thus, we visualized the normalized counts of genes belonging to the biologically interesting functional categories to see how the expression changes in the individual mouse strains (Figure 4). As described above, the classical MHC I gene *H2‐D1* showed a significantly higher expression in mock‐infected B6 mice compared with mock‐infected SJL mice (Table S2). The copy numbers of the second class Ia gene, *H2‐K1*, were also slightly higher in mock‐infected B6 animals, but the difference was below the chosen cut‐off for log2FC (log2FC: 0.85). Following TMEV‐infection, both genes were upregulated in both mouse strains in a similar profile. Expression was higher in TMEV‐infected B6 mice compared with infected SJL mice at 4 dpi (log2FC: 1.37 and 1.21 for H2‐D1 and H2‐K1, respectively) and almost equal in TMEV‐infected SJL and B6 mice at 7 and 14 dpi (log2FC < 0.3). In addition to the classical MHC I genes, several nonclassical MHC I genes showed differential expression profiles in the experiment. Six of those genes showed higher upregulation in B6 mice during early stages of the infection (similar to that of *H2‐D1*), while four others were upregulated to higher levels in SJL animals. The detailed expression profiles of all MHC genes are depicted in Figure S3. Besides MHC I genes, TMEV‐infected B6 mice showed an earlier and stronger upregulation of several genes typically expressed by natural killer (NK) cells or associated with their activation and proliferation (e. g. *Ncr1*, several killer cell lectin‐like receptors, *Klf12, Elf4, Slamf7*), in comparison TMEV‐infected SJL mice (Figure 4, Table S2). The same was true for other genes associated with innate antiviral immune responses, including two Toll‐like receptor genes (*Tlr8* and *Tlr9*), four genes of the Tripartite motif‐containing protein (Trim) family (*Trim12a, Trim12c, Trim30c, and Trim30d*), and several other interferon‐induced genes (*Ifi206, Ifi208, Ifi209, Ifi211, Ifi213, Gbp6, Tgtp2, and Mndal*, Figure 4, Table S2). The maximal discrepancy between TMEV‐infected SJL and B6 mice was observed in the expression of the antiviral protein *Trim12a* with a log2FC ranging from 4.4 to 5.1 (Figure 4, Table S2). Moreover, B6 mice showed a constitutive high expression of *Rnasel*. Although SJL mice upregulated *Rnasel* transcription following TMEV‐infection compared with mock‐infected animals, expression did not reach the level found in the brain of mock‐ or TMEV‐infected B6 animals (Figure 4, Table S2).

### 3.5 Theiler's murine encephalomyelitis virus‐infected SJL mice show an elevated transcription of MHC II and other pro‐inflammatory genes in the brain

The number of genes showing a higher expression in TMEV‐infected SJL mice compared with infected B6 mice was considerably smaller. Grouping of genes resulted in six clusters with similar expression profiles (clusters 4, 5, and 7 to 10, see Figure 3 and Figure S2). Most genes in cluster 4 showed no significant differences between SJL and B6 mice in noninfectious conditions, but a higher absolute expression in infected SJL mice at 4 or 7 dpi. Enrichment analysis revealed that the term *chemokine‐mediated signaling pathway* was significantly overrepresented in this cluster (Table 1). The mean log2FC in Cluster 5 was below −1.5, indicating that the majority of genes were already differentially expressed in mock‐infected animals. The difference increased following TMEV‐infection and peaked at 7 dpi. Functional annotation yielded no significantly enriched terms in this cluster, which was probably due to the fact that many transcripts in this cluster do not have an official gene symbol yet and thus are not functionally annotated. Genes in cluster 7 to 10 showed similar kinetics of the mean log2FC (Figure S2). They were already differentially expressed in mock‐infected animals, and differences increased following infection, with a peak at 4 dpi. The number of genes in these clusters was not sufficient to yield significant results in the functional annotation analysis. A manual screening of gene clusters with a higher expression in SJL mice revealed that they contained several transcripts involved in immune responses. The expression values of the most interesting genes are depicted in Figure 4.

Expression of many of those genes peaked at 7 dpi and the most significant differences were observed at that time point (Figure 3). Among them, four genes were involved in MHC II‐mediated antigen processing and presentation (*H2‐Ob*, *H2‐DMb2*, *H2‐Eb*, *Ctse*, see also Figure S3). The list also included genes encoding for components of the complement system or associated with complement activation (*C1s2, C1rb, C4a, and Masp2*), chemotactic factors (*Cxcl11, Cxcl13, Ccr1, Ccr9, Ccl8, and Ccl21a*) and a few interferon‐induced genes (*Gbp8, Gbp2b, and Ifi202b*). Among the latter, the most profound difference was observed in the expression of *Ifi202b* (log2FC −3.1 to −5.6). *Ifi202b* was also among the top 10 most upregulated genes in TMEV‐infected SJL mice compared with mock‐infected SJL mice (log2FC: 3.9 to 5.1, see Figure S1). Another group of genes with a higher expression in the SJL strain contained genes typically highly expressed by Tregs, such as *Ctla4, Icos, Izumo1r, and Il10* (Figure 3, Table S2). The Treg‐specific transcription factor *Foxp3* also showed a significantly higher expression in SJL mice at 7 dpi, but the difference did not reach the chosen cut‐off criterion for the fold change (log2FC: −1.2, padj: 0.005). TMEV‐infected SJL mice also showed more transcripts of the murine plasma cell marker *Ly6k*, when compared with TMEV‐infected B6 mice (Figure 3, Table S2).

### 3.6 Immunohistochemistry confirms differences observed on the transcriptomic level

**FIGURE 5:**
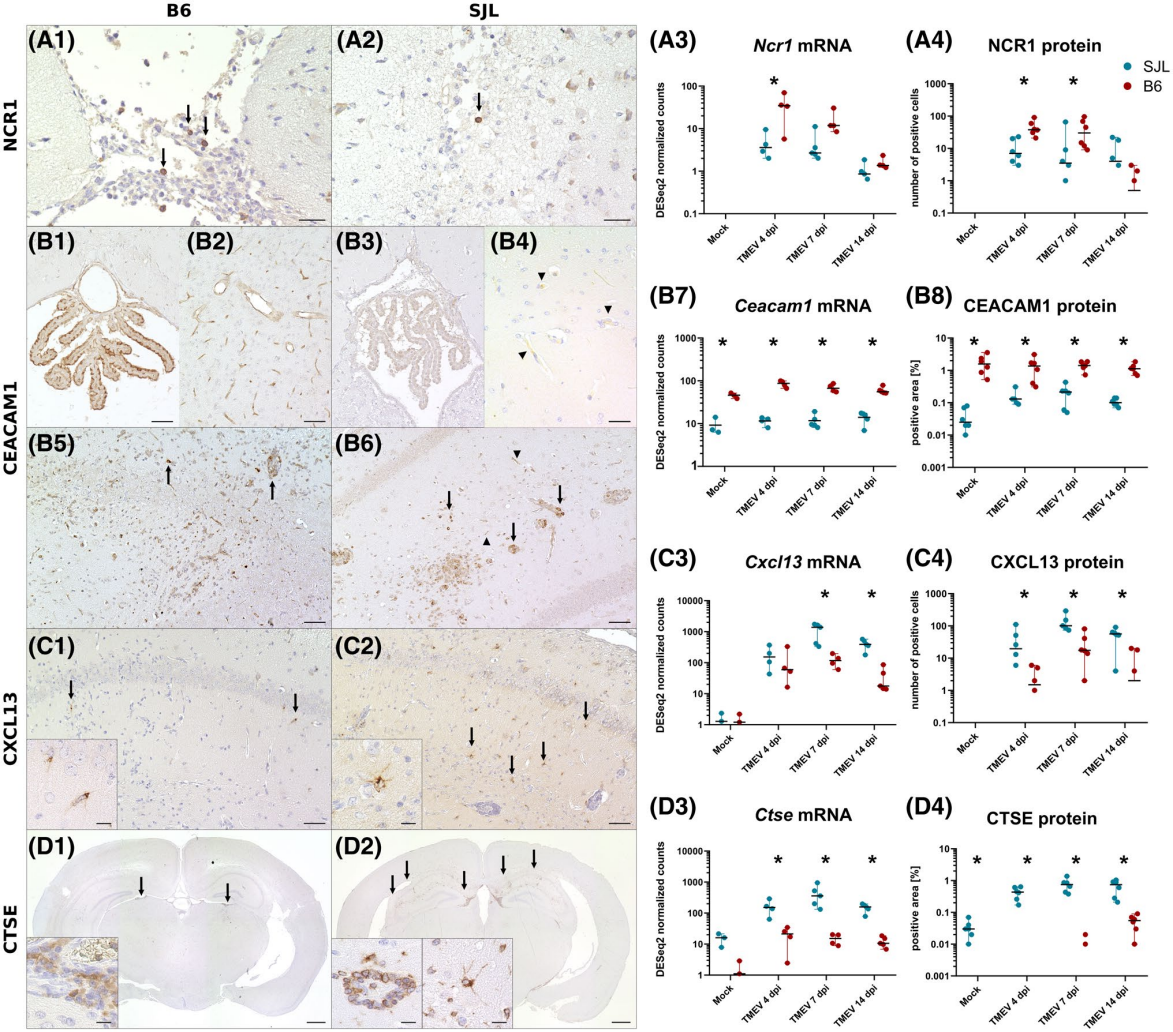
Quantification of protein products of selected genes with differential expression between SJL and B6 mice by immunohistochemistry. (A1 and A2) Immunohistochemistry (IHC) for natural cytotoxicity triggering receptor 1 (NCR1, arrows) in TMEV‐infected B6 and SJL mouse at 4 dpi. (A3 and A4) Quantification of Ncr1 mRNA and protein. (B1–B6) IHC for carcinoembryonic antigen‐related cell adhesion molecule 1 (CEACAM1) in mock‐infected (B1‐B4) and TMEV‐infected B6 and SJL mice at 7 dpi (B5 and B6). Under noninfectious conditions, B6 mice express CEACAM1 in choroid plexus cells (B1) and endothelial cells (B2), while only weak expression in some endothelial cells is detected in mock‐infected SJL animals (B4, arrowheads). Following TMEV‐infection, CEACAM1 is detected in infiltrating inflammatory cells in B6 and SJL mice (B5 and B6, arrows). Infected SJL mice also show increased immunolabeling of endothelial cells (B6, arrowheads). (B7 and B8) Quantification of Ceacam1 mRNA and protein. (C1 and C2) IHC for CXCL13 in TMEV‐infected B6 and SJL mouse at 7 dpi showing immunolabeled cells with a glial morphology. (C3 and C4) Quantification of Cxcl13 mRNA and protein. (D1 and D2) IHC for cathepsin E (CTSE) in TMEV‐infected B6 and SJL mouse at 14 dpi. In B6 mice, CTSE expression is only detected in a few perivascular inflammatory cell aggregates (D1, arrows and insert). SJL mice express abundant CTSE in different cell types, including perivascular leukocytes and glial cells (D2, arrows and inserts). (D3 and D4) Quantification of Ctse mRNA and protein. Scale bars: A1, A2, B4: 25 µm, B1–B3, B5–C2: 50 and 10 µm (inserts in C1 and C2), D1, D2: 500 and 10 µm (inserts). The graphs depict DESeq2 normalized counts of mRNA determined by RNA‐seq of cerebral tissue (A3, B7, C3, D3) and quantification of the respective protein by immunohistochemistry performed on complete transverse section of the cerebrum (A4, B8, C4, D4). For quantification of NCR1 and CXCL13, positive cells were counted, while CEACAM1 and CTSE protein levels were determined by morphometry. Lines show median and range. *Significant difference (Mann–Whitney U‐Test, *p* < 0.05). n = 3 (mock) or 4–5 (TMEV) animals/group/time point for RNA‐seq and n = 6 (mock and TMEV) animals/group/time point for immunohistochemistry. The graphs are generated with a log‐scale, zero‐values are therefore not plotted

To confirm selected differences detected by RNA‐seq, immunohistochemistry for products of two genes with a higher expression in TMEV‐infected B6 mice (*Ncr1* and *Ceacam1*) and two genes with a higher expression in TMEV‐infected SJL mice (*Cxcl13* and *Ctse*) was performed. Representative images of all immunostainings and details on cerebral mRNA and protein quantification are given in Figure 5.

Transcriptome analysis revealed that genes involved in NK cell activation were overrepresented among the genes showing an enhanced upregulation in B6 mice compared with SJL mice in early TMEV infection. NK cells have been previously proposed to be crucially involved in virus control in the TMEV model (58, 59). Of note, previous investigations have used NK1.1 as a marker for NK cells, which is known to be also expressed by some T cells. Currently, the cell surface receptor NCR1 (also known as NKp46) is considered the most specific marker for NK cell in mice and other mammals (60, 61). Following TMEV‐infection, multifocal groups of NCR1^+^ cells with lymphocyte morphology and a distinct membranous immunostaining were detected in B6 mice (Figure 5A1). The cells were found predominantly within the meninges and the choroid plexus of the third and the lateral ventricles. Labeled parenchymal cells were rarely detected. In the majority of TMEV‐infected SJL mice, only a few scattered cells were found in similar locations (Figure 5A2). The absolute number of NCR1^+^ cells within the cerebrum was significantly higher in TMEV‐infected B6 mice compared with infected SJL mice at 4 and 7 dpi (*p*‐values: 0.006 and 0.030), and decreased to low levels at 14 dpi, largely reflecting the differences observed on transcriptional level (Figure 5A3,A4). No NCR1^+^ cells were detected in brain tissues of mock‐infected animals of any mouse strain.

Another gene that showed a significantly enhanced expression in B6 mice compared with SJL mice following TMEV infection was *Ceacam1*. CEACAM1 is a cell‐cell adhesion molecule expressed by leukocytes, epithelial, and endothelial cells, which is involved in modulation of innate and adaptive immune responses (62). In CD8^+^ T cells, it is essential for activation and has been shown to improve control of chronic CNS infection (62). Since CD8^+^ T cell responses are crucial for the clearance of TMEV, we sought to determine whether an enhanced expression of CEACAM1 is present in B6 mice. In the brain of mock‐ and TMEV‐infected B6 mice, a strong CEACAM1 labeling was constantly present on vascular endothelial cells and choroid plexus epithelium (Figure 5B1,B2). In mock‐infected SJL animals, a weak positive signal was only inconstantly detected on endothelial cells (Figure 5B3,B4). Following TMEV‐infection, CEACAM1 was additionally expressed in infiltrating mononuclear cells in the brain of B6 mice (Figure 5B5). A similar proportion of immunolabeled leukocytes were detected in TMEV‐infected SJL mice, and the cells showed comparable signal intensities. Thus, the differences observed between B6 and SJL animals are probably not the result of differential expression of *Ceacam1* in T cells. In addition, an increased immunostaining was observed on endothelial cells of TMEV‐infected SJL mice in areas of neuroinflammation (Figure 5B6). Quantification of *Ceacam1* mRNA and protein expression showed almost constant levels in mock‐ and TMEV‐infected B6 mice (Figure 5B7,B8), probably due to the constitutive expression in endothelial and plexus epithelial cells, which masked the increase of infiltrating CEACAM1^+^ leukocytes. In SJL mice, the mRNA levels were also fairly constant, but protein levels increased following TMEV‐infection. Transcript and protein levels were significantly different between B6 and SJL mice at all investigated time points (*p*‐values IHC: 0.004 in mock‐infected animals and 0.006, 0.004, and 0.028 in TMEV‐infected animals at 4, 7, and 14 dpi, respectively).

Next, we looked at the distribution of two gene products which showed increased transcription in SJL mice compared with B6 mice, namely *Cxcl13* and *Ctse*. CXCL13 is a B cell chemoattractant, which is expressed in the CNS by various cell types in different pathological conditions (63, 64, 65, 66). B cell responses are suspected to contribute to demyelination in MS and its animal models, and elevated levels of B cell supporting cytokines have been detected in the CSF and the spinal cord during the chronic phase of TMEV‐infection (64, 67, 68, 69). Therefore, we sought to evaluate whether the expression is already elevated in acute infection and determine the distribution of cells producing the chemokine. CXCL13 protein was detected in the cytoplasm of glial cells within inflammatory foci in the hippocampus and other brain regions of TMEV‐infected mice. Moreover, CXCL13^+^ spindeloid or ramified cells were found admixed with perivascular and meningeal inflammatory cell aggregates in various locations. Despite a comparable extent and severity of inflammation, only a few immunolabeled cells were detected in TMEV‐infected B6 mice, while SJL mice showed numerous CXCL13^+^ cells (Figure 5C1,C2). Statistical analysis revealed a significant difference in the number of positive cells at all investigated time points following infection (Figure 5C3,C4, *p*‐values: 0.036, 0.011, and 0.012 at 4, 7, and 14 dpi, respectively). No CXCL13 protein was detected in mock‐infected animals of any mouse strain.

Another finding of RNA‐Seq analysis was the strong upregulation of genes involved in antigen presentation via MHC II in SJL mice. Since type IV hypersensitivity responses are involved in the pathogenesis of chronic demyelinating disease in this mouse strain, we sought to investigate the dynamics of MHC II responses on a protein level (8). Since there are currently no antibodies available targeting MHC molecules from animals on an H2^s^ background (e. g. SJL mice), we chose to investigate the expression of CTSE (cathepsin E), which is a protease involved in the processing of peptide antigens for presentation via MHC II (70). Immunohistochemistry detected only a few foci of immunolabeled, perivascular cells in two and six TMEV‐infected B6 mice at 7 and 14 dpi, respectively (Figure 5D1), while no protein expression was found in mock‐infected animals of that mouse strain. By contrast, abundant CTSE protein was detected in various areas of the forebrain of TMEV‐infected SJL mice, including perivascular inflammatory cells and cells with glial morphology (Figure 5D2). In mock‐infected SJL mice, some glial cells in vicinity of the injection site also showed positive staining. Accordingly, morphometric analysis resulted in significantly different protein levels between SJL and B6 mice at all investigated time points (Figure 5D3,D4, *p*‐values 0.002 in mock‐infected animals and 0.002, 0.003, and 0.007 in TMEV‐infected animals at 4, 7 and 14 dpi, respectively).

In summary, immunohistochemistry confirmed the quantitative data obtained by RNA‐seq analysis. In addition, the results provided further information on the distribution of the investigated proteins in noninfected and TMEV‐infected CNS tissue of B6 and SJL mice.

### 3.7 In additional to absolute differences in expression, many innate immune genes show different kinetics in SJL and B6 mice following virus infection

The performed pairwise comparisons of gene expression between B6 and SJL mice at different time points post infection focused on the detection of genes that show marked absolute differences between the strains. However, biological processes can be governed by small but coordinated changes in gene expression as well, which will be missed by employing stringent cut‐off criteria, such as high‐fold changes. Moreover, the timing of transcriptional changes that influence host responses can have a major impact on the course of infectious diseases. In order to dissect whether certain genes or pathways show markedly different kinetics in B6 and SJL mice over the course of infection, we performed an additional time course analysis employing the likelihood ratio test from the DESeq2 package in R (cut‐off of *p* < 0.01). This test compares relative changes in gene expression that occur in SJL and B6 mice at different time points after TMEV infection in respect to noninfectious conditions. This means that genes showing a parallel up‐ or down‐regulation in both mouse strains will not result in a low *p*‐value, even if the difference in the absolute gene expression is statistically significant. Therefore, the list of DEGs detected by the method is expected to differ from the one obtained by pairwise comparisons. Time course analysis detected 612 DEGs (Table S4). Functional annotation of these genes showed an overrepresentation of gene sets related to *RNA processing, neurogenesis, protein transport*, and mitochondrial functions, but yielded no categories specifically associated with immune responses (Table S5).

Comparison of DEG lists obtained by pairwise comparisons and by time course analysis showed that 65 genes were detected by both methods, indicating that these genes show differential expressions over time as well as high absolute differences between SJL and B6 mice. About 53 of these genes belonged to cluster 1 and 2 of DEGs obtained by pairwise comparisons (see Figure 3), which showed an early and strong upregulation in B6 mice at 4 days post TMEV infection. Among these were the genes *H2‐D1, H2‐Q5, H2‐Q2, RnaseL, Tlr8, Tlr9, Ccr2, Gzma, Klri2,* *and Klf12* (for full list see Table S4). Only seven genes with a higher expression in SJL mice were detected by both analyses and among those, the only gene associated with immune responses was the interferon inducible gene *Gbp8*.

In summary, time course analysis did not detect additional immune pathways potentially contributing to the pathogenesis of TMEV infection. However, it showed that B6 and SJL mice show significantly different kinetics in the expression of certain innate immune genes, strengthening the hypothesis that these genes could play a key role in the outcome of TMEV infection.

## 4 DISCUSSION

TMEV infection induces an acute, transient, and subclinical polioencephalitis in SJL and B6 mice. The acute phase is followed by virus elimination in B6 animals and virus persistence and demyelination in the spinal cord of SJL mice. In the presented study, the number of infected cells and degree of inflammation during acute polioencephalitis was similar in both mouse strains. However, RNA‐seq analysis of cerebral tissue revealed quantitative differences in the expression of several genes and pathways involved in immune responses and immunoregulation.

The highest number of genes with significantly different expression was detected at an early stage of infection (4 dpi), which correlated with the highest viral load in the cerebrum. Most genes in those categories showed a higher expression in B6 compared with SJL mice. Functionally, many genes were related to antigen processing and presentation via MHC I, natural killer cell activation, and innate antiviral immune responses. As described above, classical MHC I (MHC Ia) genes and MHC I‐restricted CD8^+^ T cells are particularly important for sterilizing immunity in TMEV‐infection (28). B6 mice mount a particularly protective H2‐D^b^‐restricted CD8^+^ T cell response, while SJL mice show a delayed and apparently inefficient H2‐K^s^‐restricted CTL repertoire (29, 30, 32, 33, 34, 35, 71). Boosting of H2‐K‐restricted CD8^+^ T cell responses in SJL mice results in protection from TMEV‐IDD (72). This suggests that the magnitude and timely development of CD8^+^ T cell responses, in addition to the epitope repertoire, determines the efficacy of virus clearance. In the presented data set, *H2‐D* and *H2‐K* transcripts were upregulated following TMEV‐infection in both mouse strains, but SJL mice showed lower expression of both genes in noninfectious conditions and at 4 dpi. *H2‐D1* was one of the genes that showed a significantly different absolute and relative expression between the two mouse strains. The time‐lag in MHC Ia upregulation could be a causative factor for the delayed generation of protective CTL responses in SJL mice. A recent study has elegantly demonstrated that CD11c^+^ microglia are crucially involved in the priming of protective CD8^+^ T cell responses in B6 mice, while LysM^+^ macrophages apparently play a minor role (73). It would be interesting to determine, which type of APCs is involved in the generation of CTLs in SJL mice in order to elucidate the mechanism underlying the ineffective antigen presentation in this genetic background. Moreover, analysis of transcriptomic changes in SJL mice with boostered H2‐K‐restricted CD8^+^ responses would help to dissect which changes observed in the current study are the result of insufficient CD8‐induction. For instance, it is tempting to speculate that the prolonged neuroinflammation with elevated levels of chemokines and MHC II responses in SJL mice (see discussion below) could be ameliorated or prevented in animals with timely CNS recruitment of protective CTLs. Since H2‐K‐CD8‐boostered mice show protection from disease, an analysis of their transcriptome and comparison with the presented data set could give important insight into the question which transcriptomic changes are truly involved in the pathogenesis and thus confirm causality.

In addition to classical MHC molecules, we found marked differences in the expression of several nonclassical (MHC Ib) genes in TMEV‐infected B6 and SJL mice. MHC Ib molecules are structurally similar to MHC Ia molecules but have limited or no polymorphism and serve diverse functions within and beyond the scope of immune responses (74, 75). A growing body of evidence suggests that MHC Ib‐restricted, unconventional CD8^+^ T cells participate in immune responses to viruses and bacteria, and that they can substitute for classical T cells under certain conditions (76, 77, 78, 79). Besides fostering antiviral immunity, MHC Ib molecules are also involved in immunomodulation. For instance, Qa‐1, which is encoded by the *H2‐T23* gene, induces CD8^+^ regulatory T cells (CD8^+^ Tregs). Transgenic mice bearing a mutation that specifically disrupts Qa‐1‐CD8 interaction develop a spontaneous disease characterized by autoantibody production and show exaggerated immune responses to viral CNS infection (80, 81, 82). Beneficial effects of Qa‐1‐restricted CD8^+^ Tregs have also been described in experimental autoimmune encephalomyelitis (EAE), an autoimmune model for MS (83). It is currently unknown, whether MHC Ib‐restricted CD8^+^ T cells are part of the immune response to TMEV. In the current experiment, *H2‐T23* was one of the genes showing a higher expression in B6 compared with SJL mice and could be involved in the rapid antiviral immune response or suppression of autoimmune responses by CD8^+^ Tregs. Besides *H2‐T23*, nine additional MHC Ib genes displayed a differential absolute expression and two of those (*H2‐Q2* and *H2‐Q5*) also showed significant differences in relative expression between B6 and SJL mice in the present experiment. Currently, there is little or no information on the function of these genes in the context of viral infection, so the significance of the findings remains unknown. Since MHC Ib gene products serve diverse purposes, the qualitative and quantitative differences observed in the brain of SJL and B6 mice might have consequences for the regulation of immune responses in the TMEV model.

Another group of genes that showed an increased expression in TMEV‐infected B6 mice were genes typically expressed by NK cells. A previous study in the TMEV model showed up to 50% lower NK cell activity in the spleen of SJL compared with TMEV‐IDD‐resistant C57BL/10 (B10) mice, which correlated with significantly higher viral titers in the CNS at 8 to 12 dpi (59). The present data supplements the findings by showing that besides lower activity in the periphery, SJL mice also have lower absolute numbers of NCR1^+^ NK cells in the brain during early stages of TMEV‐infection. The low NK cell number was not associated with higher viral load in the cerebrum of SJL mice but could account for the enhanced virus spread into the spinal cord. However, previous experiments have shown that NK cells are important as a first line of defense against fatal TMEV replication in the early‐phase disease, while their importance for susceptibility/resistance to demyelinating disease appears to be limited (58). NK cell depletion in TMEV‐infected B10 mice using anti‐NK1.1 or anti‐asialo‐GM1 antibodies resulted in increased clinical symptoms and lethality during acute disease but failed to induce demyelination (58, 59). Moreover, F1 crosses between SJL and B10 mice, which are “high” NK cell responders, also develop chronic disease (58, 84, 85). Of note, NK1.1 is also expressed by T cells, and previous experiments using the marker for characterization and depletion of NK cells need to be interpreted with caution (61). Currently, NKp46, encoded by the *Ncr1* gene, is considered to be a more specific marker for murine NK cells (60, 61).

B6 mice also displayed an earlier and stronger upregulation of many innate antiviral genes, including *Rnasel, Tlr8, Tlr9*, as well as genes of the *Ifi200* and *Trim* family. RNase L is an endoribonuclease that cleaves single‐stranded (ss) RNA as the last step of an interferon‐regulated, innate immune pathway (86, 87). The protective properties of the enzyme are based on direct degradation of viral RNAs and indirect antiviral mechanisms resulting from cleavage of cellular RNAs (88, 89). A recent finding has indicated that the association of the inactive, monomeric form of RNase L with cytoskeletal proteins prevents viral particle entry into the cell: the first observation of a noncatalytic function of RNase L (90). Interestingly, TMEV possesses a protein that binds RNase L and prevents its activation (91, 92). L*, encoded by an alternative reading frame unique to TMEV, is essential for virus persistence *in vivo*, presumably by facilitating survival in macrophages and microglial cells (93, 94, 95, 96). The effect of the protein is not observed in neuronal cell lines, and L* presence or absence does not influence acute disease, in which neurons are the main targets of the virus (94, 97). In the presented experiment, a significant difference was observed in the cerebral expression of *Rnasel* between SLJ and B6 mice (up to three‐fold difference) in noninfectious and infectious conditions. It is tempting to speculate that the expression and activity levels of RNase L influence virus replication and persistence in macrophages/microglial cells and thereby represents a potential determinant of susceptibility to chronic infection. Structural studies of human RNase L predict that even a two‐fold increase of expression could result in a 32‐fold increase in activity (98). TMEV replicates at a higher rate in dendritic cells derived from SJL mice than in those from B6 mice (44). It is imaginable that the higher basal expression of RNase L in B6 mice enables infected cells to overcome the inhibitory effects of L*, resulting in lower viral loads and ultimately virus clearance. Alternatively, a lower level of viral replication as consequence of other, yet unknown factors in B6 mice might be manageable for the basal RNase L activity. Besides purely quantitative effects, the differences of RNase L expression might also result in activation of different downstream effects, which was previously shown by comparison of cell types with different levels of RNase L activity (99). Further investigations regarding the cellular distribution and activity of RNase L in both mouse strains are needed to clarify the impact of the observed differential expression for TMEV persistence.

Tripartite motif (TRIM) proteins have diverse cellular functions and are currently increasingly recognized as important innate antiviral factors (100). In primates, TRIM5 restricts replication of HIV and other retroviruses and has therefore been extensively studied (101, 102, 103). In contrast to primates, which have only one *TRIM5* gene, the corresponding *Trim* locus in mice contains more than eight orthologues. The genes have apparently evolved under positive selection, which is suggestive of their significance for antiviral responses (104). However, the exact viruses putatively restricted by the murine genes are largely unknown. An antiviral function has been demonstrated in the case of *Trim30d* (encoding TRIM79α). TRIM79α restricts replication of tick‐borne encephalitis virus and Langat virus in murine macrophages by mediating lysosomal degradation of NS5, a viral RNA‐dependent polymerase (105). In addition to direct interference with the viral replication cycle, members of the Trim family also regulate signal transduction pathways induced by innate immune sensors and thereby modulate antiviral responses (100, 106). For instance, TRIM12C associates with TRAF6 and stimulates type I IFN and NF‐κB pathways (107). *Trim12a* is also a candidate gene associated with resistance to H5N1 influenza infection in mice (108). Involvement of Trim proteins in antiviral immunity to TMEV has not been investigated so far. In the current study, we found a higher expression of *Trim12a, Trim12c, Trim30c,* *and Trim30d* in B6 mice compared with SJL mice already under noninfectious conditions, and the difference increased even further upon TMEV‐infection. The differences were most striking regarding *Trim12a*, which was among the top 20 DEGs in mock‐infected animals (approximately seven‐fold difference in expression) and showed the highest difference of all DEGs in TMEV‐infected animals at 4 and 7 dpi (up to 32‐fold). Therefore, involvement of Trim proteins in responses to TMEV‐infection certainly warrants further investigation.

Besides MHC I, genes involved in antigen presentation via MHC II were significantly enriched in the list of DEGs and differences were most prominent at 7 dpi. All genes showed a higher expression in SJL mice compared with B6 animals. The role of MHC II in the TMEV model is complex and partially dependent on the disease phase. MHC II‐mediated CD4^+^ T cell responses are required for virus control, because B6 mice deficient in CD4 or MHC II show virus persistence (109, 110, 111). On the other hand, Th1‐mediated hypersensitivity to viral epitopes contributes to TMEV‐IDD progression in the chronic phase (18, 110). We chose to investigate the expression of CTSE, which is a protease involved in the processing of peptide antigens for presentation via MHC II (70). It is known that B6 mice have a cell type‐specific deficiency of CTSE in hematopoietic cells, while protein levels in other tissues (e. g., stomach) are normal in the animals (112). CTSE expression has also been previously reported in microglial cells of B6 mice with ischemia‐induced hippocampal neuronal damage (113). However, we did not detect CTSE^+^ glial cells in TMEV‐infected B6 animals using immunohistochemistry. By contrast, a widespread and abundant immunolabeling was detected in leukocytes and glial cells of TMEV‐infected SJL mice. Importantly, the protein was also expressed in areas without viral antigen and in vicinity of the injection site in mock‐infected animals. It is tempting to speculate that the protease could be involved in the presentation of endogenous antigens released in the course of CNS damage, and thereby trigger epitope spreading and autoimmunity. Conversely, the lack of CTSE expression in the CNS of TMEV‐infected B6 mice might represent a protective mechanism against this phenomenon.

In addition to MHC II genes, SJL mice showed a prolonged elevation of several genes involved in leukocyte chemotaxis, indicating sustained neuroinflammation. The list included CXCL13, which is a chemokine involved in B cell trafficking and organization and plays an important role in neuroinflammation (63, 114). Humoral responses have been suspected to contribute to demyelination in MS and its animal models for a long time (64, 67, 68, 115). The interest has been reawakened recently by the observed clinical benefit of B cell depletion therapies in MS (116). In chronic TMEV‐IDD of SJL mice, genes involved in plasma cell differentiation and antibody production are chronically upregulated in the spinal cord (117). Moreover, high numbers of antibody‐secreting and memory B cells, elevated levels of IgG and B cell supporting chemokines are detected in the spinal cord and cerebrospinal fluid in late TMEV‐infection (64, 67, 68, 69). As chronic TMEV‐IDD is characterized by an intact blood–brain barrier, these findings are strongly indicative of a compartmentalized inflammation within the CNS with intrathecal antibody production. The specificity of these responses remains largely undetermined. A monoclonal antibody cross‐reacting with viral capsid protein VP1 and the myelin component galactocerebroside (GALC) were isolated from TMEV‐infected mice, and *in* *vivo* application of that antibody into mice with EAE exacerbated demyelination (118, 119). Additionally, it has been recently demonstrated that B cells derived from SJL mice are permissive to TMEV replication *in vitro* and that infected B cells show an upregulation of activation markers, MHC I and II, and co‐stimulatory molecules, promoting T cell activation and producing polyclonal antibodies reactive with CNS antigens (120). However, the actual contribution of autoantibodies to the demyelination process in TMEV‐IDD remains undetermined. In the present study, mRNA and protein levels of CXCL13 were upregulated very early in the infection course, and the levels were significantly higher in TMEV‐infected SJL when compared with TMEV‐infected B6 mice. Moreover, transcripts of the murine plasma cell marker *Ly6k* were markedly elevated as early as 7 dpi. This is in line with a previous report showing significantly higher numbers of B cells accumulating in the brain of acutely infected SJL compared with B6 mice (39). In addition, the distribution of CXCL13‐producing cells in the cerebrum is visualized, which has not been described in the TMEV model before. CXCL13 antigen was detected in spindeloid and ramified cells within dense leukocytic aggregates in the meninges. Similar CXCL13‐producing cells were previously described within meningeal lymphoid follicle‐like structures in the EAE model and were identified as follicular dendritic cells based on FDC‐M1 expression (115). Moreover, CXCL13‐producing cells with glial morphology were observed within the brain parenchyma. We propose that in SJL mice, TMEV‐infection drives an early and excessive CXCL13 production in various cell types, which promotes accumulation and survival of B cells in the CNS. These B cells represent additional targets for the virus and promote inflammatory demyelination via production of autoantibodies and/or MHC II‐mediated activation of pathologic T cell responses.

Transcriptome data did not reveal a statistically significant overrepresentation of genes associated with a distinct CD4^+^ T cell polarization. However, several genes constitutively expressed in Foxp3^+^ Tregs showed a significantly higher expression in SJL mice. This is in agreement with previous observations showing an early expansion of Tregs following TMEV‐infection in SJL mice (39, 41). Tregs represent a double‐edged sword in TMEV‐infection and their manipulation produces diverse and partly contrasting effects depending on the disease phase and the mouse strain. In SJL mice, Tregs interfere with antiviral immunity in the acute phase, but ameliorate demyelination in the chronic phase (40, 41). In B6 mice, sole manipulation of the Treg compartment failed to influence the course of infection (38, 41, 121). Therefore, excessive Treg function cannot fully account for differences in susceptibility to TMEV persistence. However, Tregs could be involved in hippocampal protection observed in SJL animals during the acute infection phase. Neuroprotective Treg properties have been demonstrated in several animal models of viral encephalitis, including human immunodeficiency virus, murine cytomegalovirus, West Nile virus, and Japanese encephalitis virus infection (122, 123, 124, 125, 126, 127, 128, 129). Infected SJL mice also showed higher transcripts of *Il10* in the brain, which is in line with previous observations in the model and has been shown to protect hippocampal neurons from TMEV‐induced damage (39, 130).

## 5 CONCLUSIONS

In summary, many genes involved in innate antiviral immune response showed a higher expression in TMEV‐infected B6 mice, compared with TMEV‐infected SJL mice, and differences were particularly prominent during onset of the acute polioencephalitis (4 dpi). Some of those genes were also upregulated in SJL mice upon infection, but upregulation was slower and/or weaker than that in B6 animals and peaked at 7 dpi. This time‐lag does not appear to compromise virus clearance from the cerebrum, but presumably facilitates spread into the spinal cord, which is associated with a switch in cell tropism and virus persistence in glial cells and macrophages (8, 10). Besides enabling persistent TMEV replication, the suboptimal expression of innate immune genes in SJL mice could be a causative factor for the induction of unfavorable adaptive immune responses. A stronger and prolonged upregulation of MHC II genes, chemotactic molecules, and genes associated with antibody production were observed in infected SJL mice, compared with infected B6 mice, which might play a role in the induction of delayed‐type hypersensitivity and autoimmunity. Lastly, infected SJL animals showed higher levels of immunomodulatory genes, which presumably contribute to hippocampal protection. The study confirms several previous observations in the TMEV model and expands the list of immunologic parameters potentially contributing to different outcomes in TMEV‐infected B6 and SJL mice. Besides the factors discussed above, an extensive list of genes differentially expressed between SJL and B6 mice and not previously investigated in TMEV‐infection is given, providing a broad basis for further mechanistic research in the model.

## CONFLICT OF INTEREST

The authors declare no conflict of interests.

## ETHICS APPROVAL

The animal experiments were approved and authorized by the local authorities (Niedersächsisches Landesamt für Verbraucherschutz‐ und Lebensmittelsicherheit (LAVES), Oldenburg, Germany, permission numbers: 509c‐42502‐02/589, 509.6–42502‐04/860 and 33–42502‐05/963).

## Supporting information


**FIGURE S1** Pairwise comparison of gene expression between mock‐ and Theilervirus‐infected SJL or B6 mice. Volcano plots obtained by pairwise comparisons of DESeq2 normalized counts of mock‐ or TMEV‐infected SJL and B6 mice (reference: mock) at 4, 7, and 14 days post infection (dpi). Cut‐off for differential expression was set at │log2fold change (FC)│ > 1.5 and a corrected *p*‐value of <0.05.The top 10 upregulated genes (lowest *p*‐value) in both mouse strains are listed in the tables. Venn diagrams depict number of overlapping DEGs at the three time points

**FIGURE S2** Mean log2 fold change (Log2FC) of differentially expressed gene (DEGs) sets obtained by pairwise comparison of B6 and SJL mice following mock or TMEV infection (reference: SJL). log2FC values > 0 (higher expression in B6 mice) are shown in red and log2FC < 0 (higher expression in SJL mice) in blue. Cluster numbers refer to the gene clusters with a similar expression pattern as displayed in Figure 3

**FIGURE S3** Expression profiles of differentially expressed MHC genes in the cerebrum of mock‐ and Theilervirus‐ infected SJL and B6 mice. The graphs depict DESeq2 normalized counts of mRNA determined by RNAseq of cerebral tissue. Lines show median and range. The asterisk indicates a corrected *p*‐value of <0.05, regardless of the fold change. n = 3‐5 animals/group/time point

**TABLE S1** Correlation analysis of TMEV‐ antigen, positive, and negative stranded RNA

**TABLE S2** Differentially expressed genes derived by pairwise comparison of B6 and SJL mice following mock‐ or TMEV infection

**TABLE S3** Overrepresentation analysis of gene sets with a differential expression between mock‐ or TMEV‐infected SJL and B6 mice (pairwise comparisons)

**TABLE S4** Differentially expressed genes obtained by time course analysis of B6 and SJL mice following mock‐ or TMEV infection

**TABLE S5** Overrepresentation analysis of differentially expressed genes obtained by time course analysis

## Data Availability

RNA‐seq data can be accessed at GEO/SRA (https://www.ncbi.nlm.nih.gov/geo/) under accession number GSE159226 (https://www.ncbi.nlm.nih.gov/geo/query/acc.cgi?acc=GSE159226). All other datasets generated during the current study are available from the corresponding author on reasonable request.
