# Supplementary figures and images for: Transcriptome analysis following neurotropic virus infection reveals faulty innate immunity and delayed antigen presentation in mice susceptible to virus‐induced demyelination
Source: Brain Pathol. 2021 Jul 6;31(6):e13000. doi: 10.1111/bpa.13000 (PMC8549031; doi:10.1111/bpa.13000)

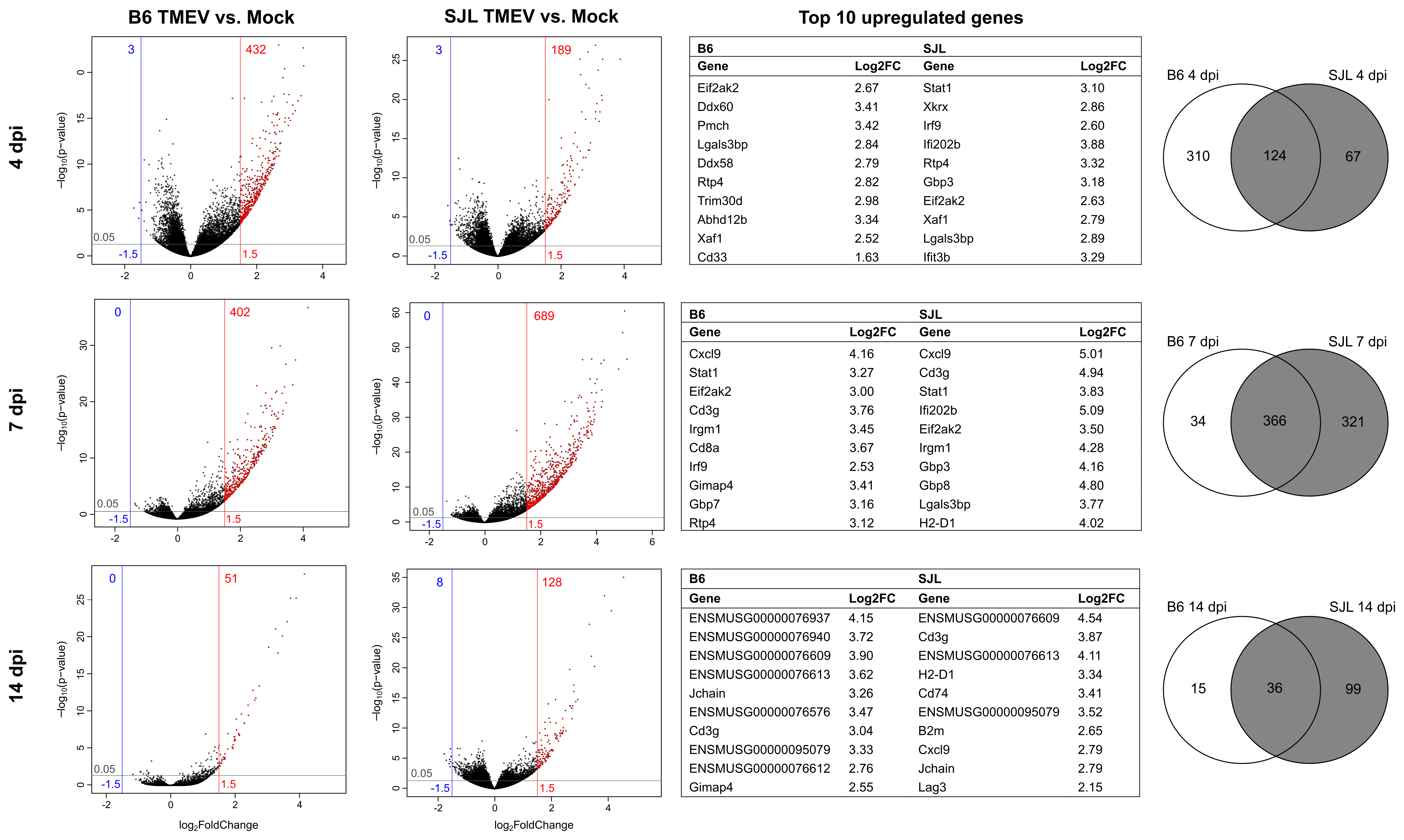

Supplement: Supplementary file 1 — FIGURE S1 Pairwise comparison of gene expression between mock‐ and Theilervirus‐infected SJL or B6 mice. Volcano plots obtained by pairwise comparisons of DESeq2 normalized counts of mock‐ or TMEV‐infected SJL and B6 mice (reference: mock) at 4, 7, and 14 days post infection (dpi). Cut‐off for differential expression was set at │log2fold change (FC)│ > 1.5 and a corrected p‐value of <0.05.The top 10 upregulated genes (lowest p‐value) in both mouse strains are listed in the tables. Venn diagrams depict number of overlapping DEGs at the three time points [file BPA-31-e13000-s008.png]

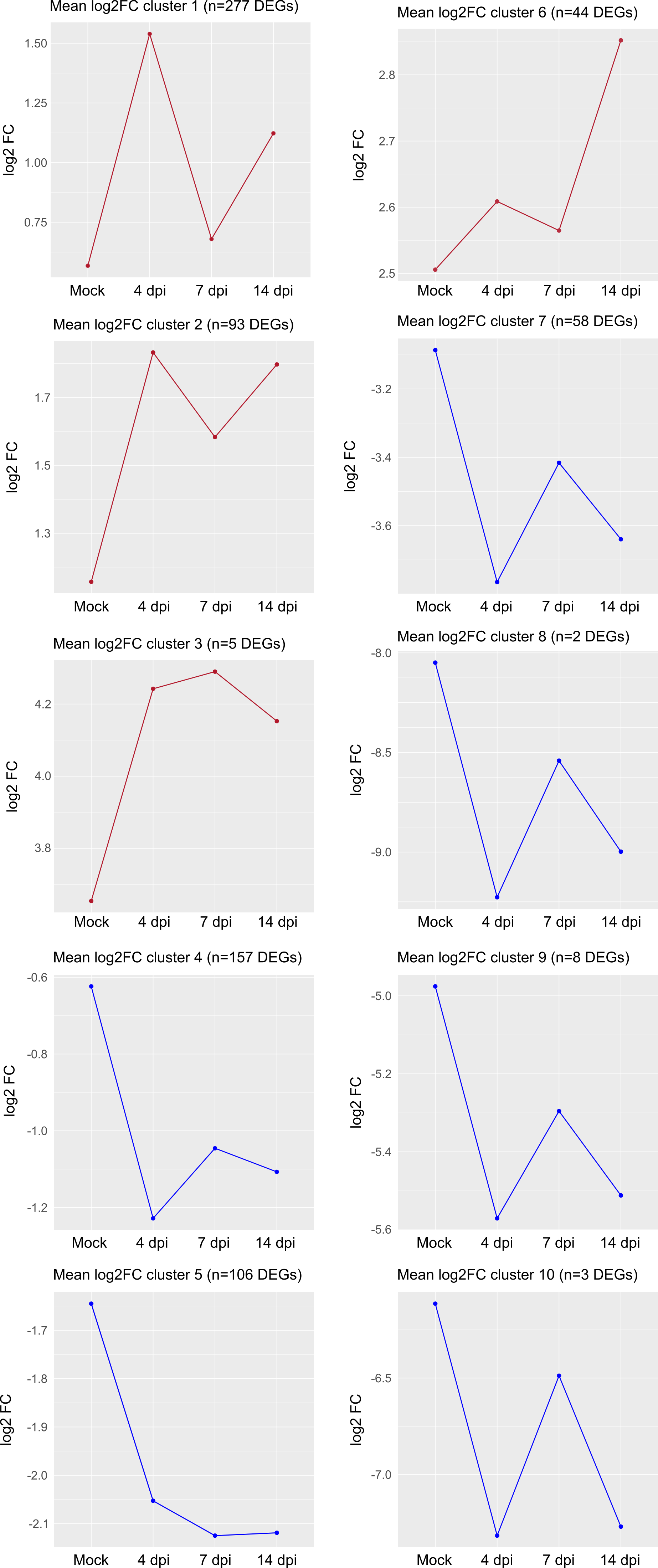

Supplement: Supplementary file 2 — FIGURE S2 Mean log2 fold change (Log2FC) of differentially expressed gene (DEGs) sets obtained by pairwise comparison of B6 and SJL mice following mock or TMEV infection (reference: SJL). log2FC values > 0 (higher expression in B6 mice) are shown in red and log2FC < 0 (higher expression in SJL mice) in blue. Cluster numbers refer to the gene clusters with a similar expression pattern as displayed in Figure 3 [file BPA-31-e13000-s001.png]

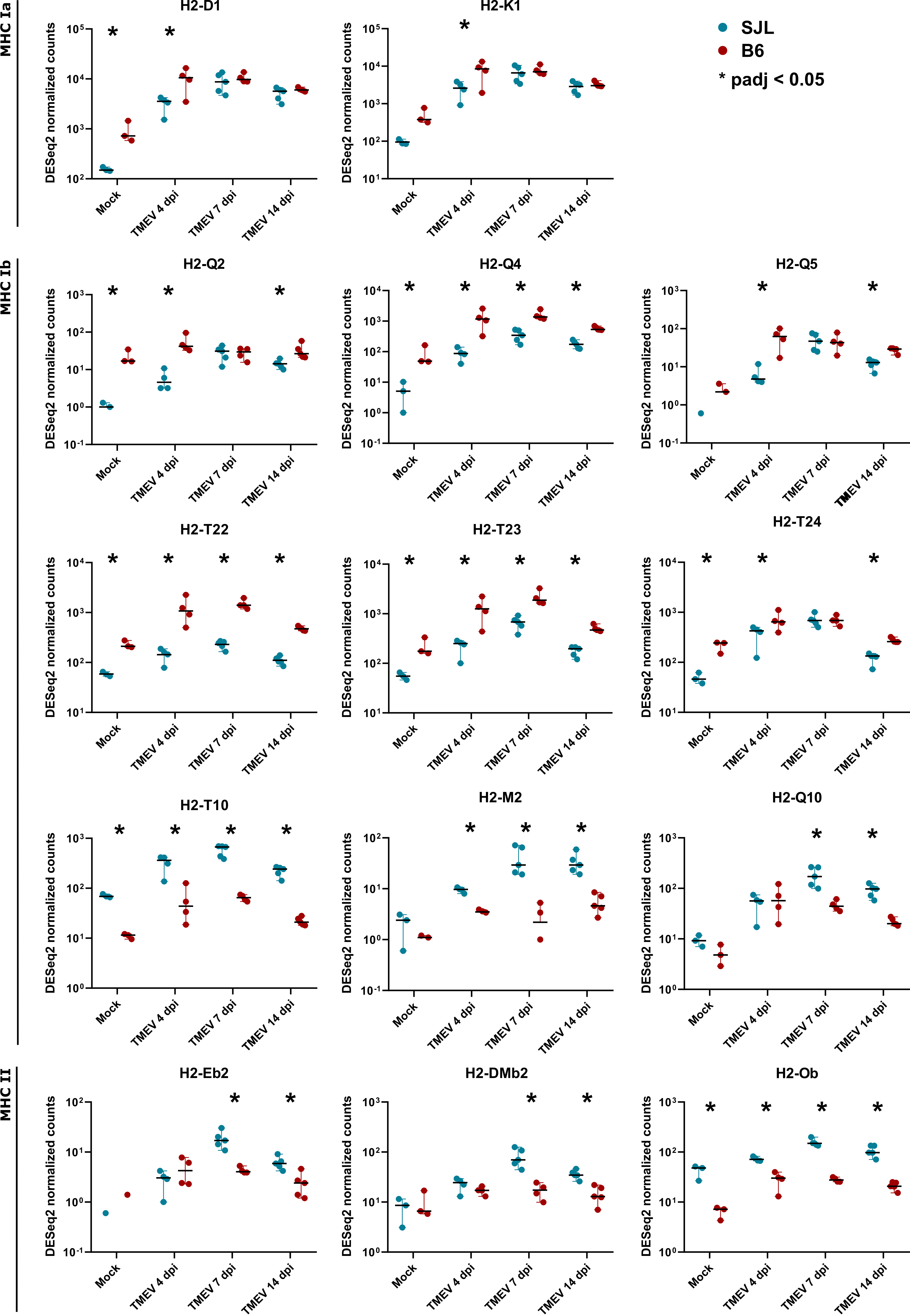

Supplement: Supplementary file 3 — FIGURE S3 Expression profiles of differentially expressed MHC genes in the cerebrum of mock‐ and Theilervirus‐ infected SJL and B6 mice. The graphs depict DESeq2 normalized counts of mRNA determined by RNAseq of cerebral tissue. Lines show median and range. The asterisk indicates a corrected p‐value of <0.05, regardless of the fold change. n = 3‐5 animals/group/time point [file BPA-31-e13000-s004.png]
