# Supplementary material for: Transcriptome analysis following neurotropic virus infection reveals faulty innate immunity and delayed antigen presentation in mice susceptible to virus‐induced demyelination
Source: Brain Pathol. 2021 Jul 6;31(6):e13000. doi: 10.1111/bpa.13000 (PMC8549031; doi:10.1111/bpa.13000)
Supplement: Supplementary file 4 — TABLE S1 Correlation analysis of TMEV‐ antigen, positive, and negative stranded RNA [file BPA-31-e13000-s005.docx]

**Supplementary file S1:** Correlation analysis of TMEV- antigen, positive and negative stranded RNA

| **Correlation** | **Cerebrum** | | **Spinal cord** | |
| --- | --- | --- | --- | --- |
|  | **r_s_** | **p** | **r_s_** | **p** |
| Antigen and (+)RNA | 0.882 | <0.0001 | 0.871 | <0.0001 |
| Antigen and (-) RNA | 0.686 | <0.0001 | 0.558 | <0.0001 |
| (+) RNA and (-) RNA | 0.732 | <0.0001 | 0.733 | <0.0001 |
